# Low Levels of Physical Activity Increase Metabolic Responsiveness to Cold in a Rat (*Rattus fuscipes*)

**DOI:** 10.1371/journal.pone.0013022

**Published:** 2010-09-27

**Authors:** Frank Seebacher, Elsa J. Glanville

**Affiliations:** Integrative Physiology Research Group, School of Biological Sciences, The University of Sydney, Sydney, New South Wales, Australia; Roehampton University, United Kingdom

## Abstract

**Background:**

Physical activity modulates expression of metabolic genes and may therefore be a prerequisite for metabolic responses to environmental stimuli. However, the extent to which exercise interacts with environmental conditions to modulate metabolism is unresolved. Hence, we tested the hypothesis that even low levels of physical activity are beneficial by improving metabolic responsiveness to temperatures below the thermal neutral zone, thereby increasing the capacity for substrate oxidation and energy expenditure.

**Methodology/Principal Findings:**

We used wild rats (*Rattus fuscipes*) to avoid potential effects of breeding on physiological phenotypes. Exercise acclimation (for 30 min/day on 5 days/week for 30 days at 60% of maximal performance) at 22°C increased mRNA concentrations of PGC1α, PPARδ, and NRF-1 in skeletal muscle and brown adipose tissue compared to sedentary animals. Lowering ambient temperature to 12°C caused further increases in relative expression of NRF-1 in skeletal muscle, and of PPARδ of brown adipose tissue. Surprisingly, relative expression of UCP1 increased only when both exercise and cold stimuli were present. Importantly, in sedentary animals cold acclimation (12°C) alone did not change any of the above variables. Similarly, cold alone did not increase maximum capacity for substrate oxidation in mitochondria (cytochrome c oxidase and citrate synthase activities) of either muscle or brown adipose tissue. Animals that exercised regularly had higher exercise induced metabolic rates in colder environments than sedentary rats, and temperature induced metabolic scope was greater in exercised rats.

**Conclusions/Significance:**

Physical activity is a necessary prerequisite for the expression of transcriptional regulators that influence a broad range of physiological functions from energy metabolism to cardiovascular function and nutrient uptake. A sedentary lifestyle leads to decreased daily energy expenditure because of a lack of direct use of energy and a muted metabolic response to ambient temperature, which can be reversed even by low levels of physical activity.

## Introduction

Animal health and reproductive success is largely determined by a balance between energy intake and expenditure. Too little chemical energy (adenosine triphosphate, ATP) available to cells will constrain animal function [1] and too much energy ingested will lead to obesity and disease [2]–[4]. Energy is expended to maintain cellular homeostasis, and on growth and locomotion. Physical activity is a default condition for most mammals [5] and the regulatory pathways that control ATP production have evolved with exercise as a principal selection pressure [6]–[8]. The mammalian genome may therefore be maladapted to inactivity to the extent that switching from an active to a sedentary lifestyle could disrupt metabolic signaling and cause medical conditions such as obesity, diabetes, and cardiovascular disease [9]–[12].

Aerobic, or oxidative, metabolism is the principal avenue for ATP production in all animals, and also for heat production in endotherms. In the cell, mitochondria are the site for oxidative metabolic pathways. The capacity of mitochondria to oxidise substrates derived from food is important for whole animal energy expenditure [14], but mitochondria are effective in facilitating whole body energy use only when cells are in a negative energy balance [15]. Energy is stored in mitochondria as an electrochemical proton gradient across an inner membrane in which the principal metabolic protein complexes are situated. A negative energy balance is achieved by dissipating the proton gradient across the inner mitochondrial membrane; this can occur by ATP use in the cell resulting from physical activity, which will stimulate an enzyme in the inner mitochondrial membrane (complex V or FoF1 ATPase) to replenish ATP by phosphorylating ADP using energy provided by the proton gradient. Additionally, exposure to cold will stimulate heat production in mitochondria [16] by causing protons to pass through the inner membrane without ATP production, which is facilitated by activation of specific uncoupling proteins (uncoupling protein 1 or UCP1). Both cold exposure and exercise trigger transcriptional pathways that lead to increased mitochondrial abundance and oxidative capacity in skeletal muscle and brown adipose tissue [17]–[20]. Elevated oxidative capacity, which may be reflected in the increased activity of mitochondrial enzymes such as citrate synthase and cytochrome c oxidase [17], leads to increased substrate oxidation and energy use. Regulation of mitochondrial metabolism is to a large extent achieved by a relatively small number of transcription factors and their coactivators. The transcriptional co-activator peroxisome proliferator-activated receptor gamma coactivator-1 alpha (PGC-1α) interacts with nuclear respiratory factors (NRF-1 and NRF-2) to stimulate mitochondrial biogenesis [17], [20]–[21]. Exercise also increases fatty acid oxidation in skeletal muscle by inducing peroxisome proliferator-activated receptor delta (PPARδ) expression, a transcriptional regulator of energy metabolism that plays an important role in the transcriptional regulation of muscle metabolism [8], [22]–[23]. One mechanism by which PPARδ modulates metabolism of skeletal muscle in mice is by causing a switch from glycolytic, fast muscle fibres to oxidative, slow muscle fibres, resulting in greater running performance and aerobic capacity [24]. Interestingly, however, pharmacological stimulation of PPARδ, does not necessarily improve endurance performance although it does cause an increase in transcription of some genes important in oxidative metabolism [13]. However, PPARδ stimulation in combination with physical activity induces the expression of a suite of metabolic genes, improves endurance performance, and reduces fat content, which indicates that exercise is a necessary trigger allowing PPARδ to interact with otherwise cryptic target genes [13]. These data suggest that physical activity may be required to achieve patterns of gene expression that permit effective regulation of energy balance [6], [8], [15]. Additionally, cold stimulates mitochondrial uncoupling and energy use in brown adipose tissue (BAT) by increasing the activity and transcription of mitochondrial uncoupling protein 1 (UCP1; [2], [25]–[27]).

In the absence of either cold or activity, metabolic responses will be muted, which may explain the limited metabolic response of experimental stimulation of metabolic regulators [13], [15]. Although cold exposure and exercise may each stimulate mitochondrial flux and capacity, it is not clear whether the two act independently [6]–[8], [10], [12]. Hence, it was our aim to determine whether cold exposure and physical activity interact to modulate energy metabolism.

In the context of endothermic metabolism, “cold” may be defined as an ambient temperature below the thermal neutral zone when metabolic rates increase above basal rates [2]. In practice, most endotherms experience environmental temperatures below their thermal neutral zone most of the time; for example, the thermal neutral zone of humans is 33–35°C, and that of *Rattus fuscipes* is 29–31°C. Metabolic responses triggered by ambient temperature therefore constitute a principal avenue of daily energy expenditure [16]. If, however, metabolic responses to cold are dependent on a behavioural trigger such as physical activity, the reduction in daily energy use in sedentary animals would be twofold, firstly resulting from a lack of direct exercise-related energy use, and secondly resulting from a muted metabolic response to cold.

We tested the null-hypothesis that chronic cold and regular low level exercise act independently to elicit similar metabolic responses. Hence, an animal that is sedentary and exposed to cold will have similar metabolic responses as an animal that is exercised in the warmth. The alternatively hypothesis is that there is a differential, and possibly an interactive effect of cold exposure and exercise on metabolic responses. To test these hypotheses, we exposed rats chronically to different thermal and exercise conditions in a two-factorial experimental design, and examined a functional cascade of responses from the expression of regulatory transcription factors to running performance. Our results show that metabolic responses to cold are dependent on low levels of physical activity, so that the principal avenues of daily energy expenditure are removed by a sedentary lifestyle.

## Results

### Gene Expression

**Figure 1:**
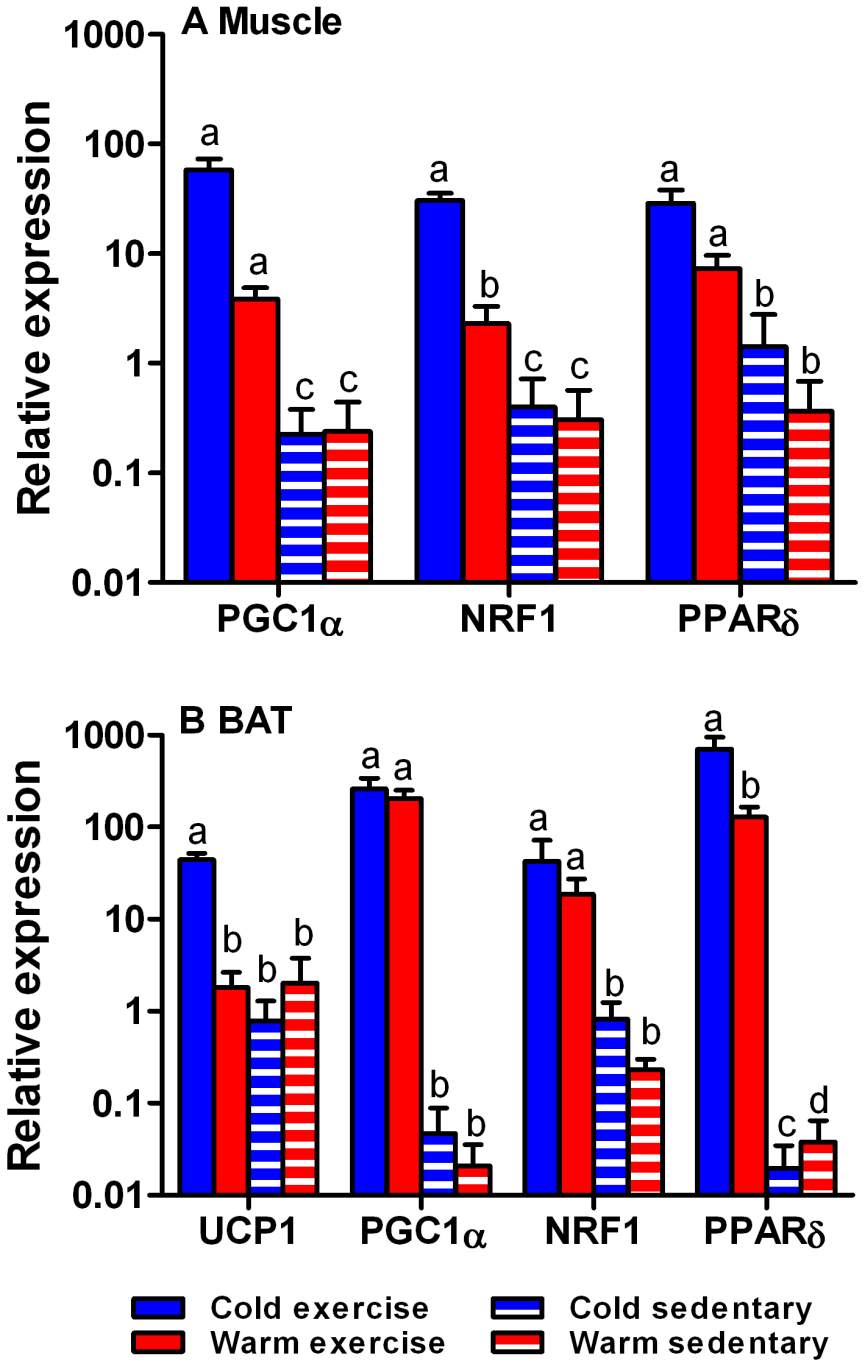
Relative expression of transcription factors and UCP1. Relative expression (mean ± s.e.m) of transcriptional regulators PGC-1α, NRF-1, and PPARδ in skeletal muscle (A) and BAT (B), and UCP1 in BAT (B) from cold exercised (blue bars), warm exercised (red bars), cold sedentary (blue striped bars) and warm sedentary (red striped bars) rats. The relative expression of all genes increased with exercise in both skeletal muscle and BAT. There were significant interactions between thermal acclimation and exercise for NRF-1 relative expression in muscle, and for PPARδ expression in BAT. UCP1 relative expression did not increase in response to either cold or exercise alone, but only when those two stimuli coincided. Significant differences are indicated by different letters above each column.

In muscle, relative expression of all target genes was significantly greater in exercised animals (main effect, all F_1,23_>55.76, p<0.0001; Fig. 1a). There was significant interaction between exercise and thermal acclimation for NRF-1 expression (F_1,23_ = 4.98, p<0.05), which means exercised and warm acclimated animals had a lower expression than exercised, cold acclimated animals (Fig. 1a). PGC-1α relative expression was lower in warm acclimated animals (F_1,23_ = 6.42, p<0.02), but there was no significant interaction between exercise and thermal acclimation (F_1,23_ = 3.45, p = 0.078). Only exercise had a significant effect on PPARδ relative expression (F_1,23_ = 55.26, p<0.0001), and neither thermal acclimation nor the interaction were significant (F_1,23_ = 3.12, p = 0.092 and F_1,23_ = 0.02, p = 0.97, respectively).

Similar to muscle, relative expression of all target genes increased with exercise treatment in BAT (main effect, all F_1,23_>15.90, p<0.0001; Fig. 1b). UCP1 mRNA levels in BAT did not increase either in response to cold or to exercise alone, but concentrations of UCP1 mRNA increased significantly when both cold and exercise stimuli were present (interaction: F_1,23_ = 9.74, p = 0.005; Fig. 1b). There was also an interaction between thermal acclimation and exercise in the relative expression of PPARδ (F_1,23_ = 4.56, p<0.05), and warm acclimated animals had lower expression when exercised but higher expression when sedentary (Fig. 1b). Contrary to expectation, cold exposure had no effect on the relative expression of PGC-1α or NRF-1 (all F_1,23_<0.5, p>0.49; Fig. 1b).

**Figure 2:**
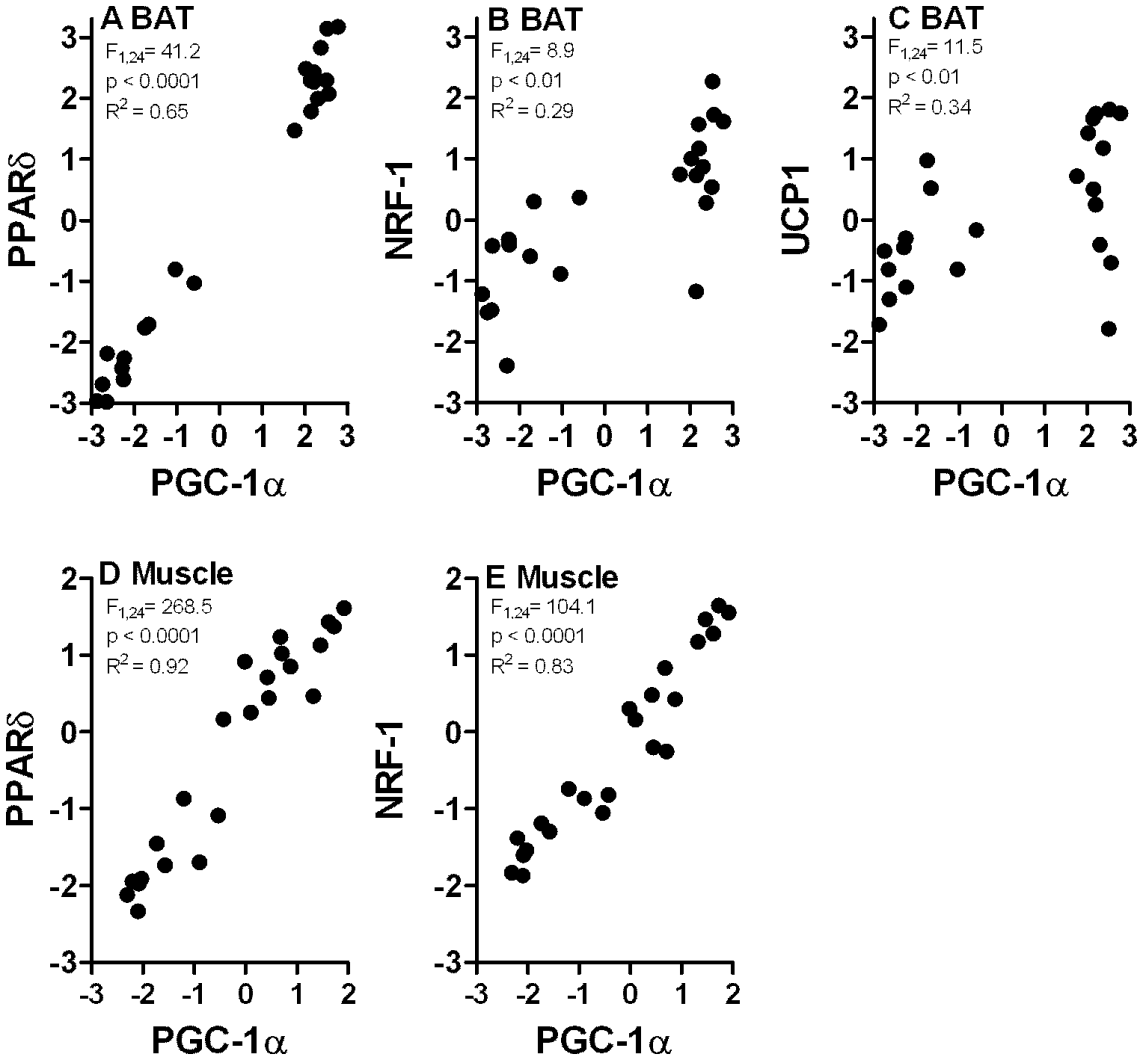
PGC-1α co-varies with PPARδ, NRF-1 and UCP1. There were significant associations between PGC-1α and PPARδ (a), NRF-1 (b) and UCP1 (c) relative expression in BAT, and between PGC-1α and PPARδ (d), NRF-1 (e) in skeletal muscle. Results form regression analyses are shown in each panel, and the axes are on a logarithmic scale.

PGC-1α relative expression was a significant predictor of PPARδ and NRF-1 relative expression in both muscle and BAT, and also of UCP1 relative expression in BAT (Fig. 2).

### Enzyme activities

**Figure 3:**
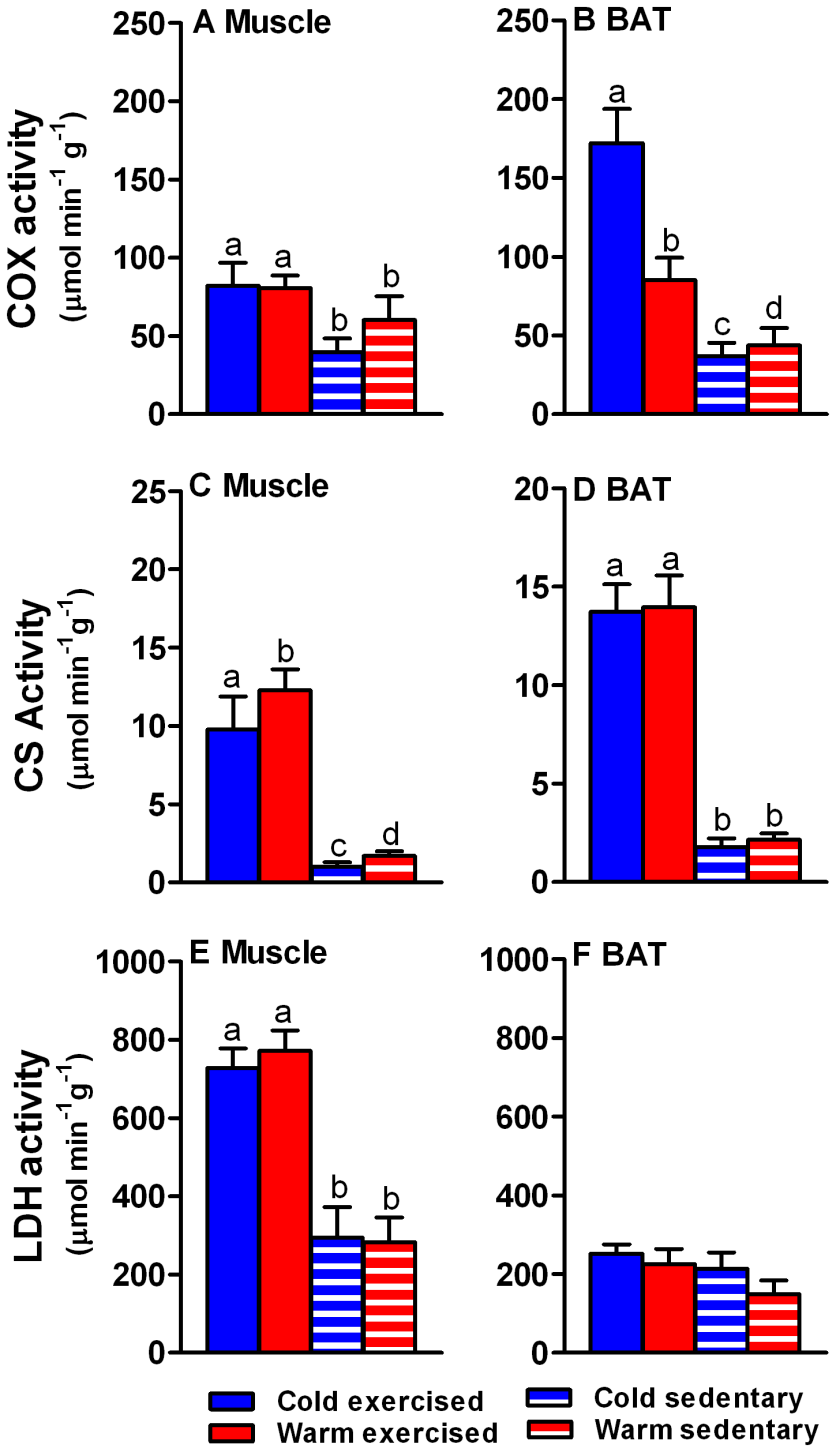
Enzyme activities in skeletal muscle and BAT from cold and warm acclimated sedentary and exercised rats. Activity (µmol substrate converted g^−1^ wet tissue; mean ± s.e.m) of cytochrome c oxidase (COX; A and B), citrate synthase (CS; C and D) and lactate dehydrogenase (LDH; E and F) of cold exercised (blue bars), warm exercised (red bars), cold sedentary (blue hatched bars) and warm sedentary (red hatched bars) rat skeletal muscle and brown adipose tissue (BAT). Exercise had a significant effect on all enzyme activities, but the effect of cold exposure alone was limited. Letters above each column indicate significant differences.

Cytochrome c oxidase (COX) activity, an indicator of the maximal capacity of mitochondria for substrate oxidation, was significantly higher in skeletal muscle (F_1,35_ = 6.16, p<0.02; Fig. 3a) of exercised rats compared to sedentary rats. Cold acclimation did not cause an change in COX activity in muscle (F_1,35_ = 0.56, p = 0.46). Similarly, COX activity in BAT of exercised rats was significantly higher than in sedentary rats (F_1,35_ = 39.86, p<0.0001), but there was an interaction between exercise and thermal acclimation indicating that cold acclimated, exercised rats had the greatest COX activity (F_1,32_ = 11.16, p<0.002; Fig. 3b).

Citrate synthase (CS) activity is a measure of mitochondrial abundance. In muscle, its activity was significantly higher in exercised (F_1,27_ = 115.84, p<0.0001) and warm acclimated rats (F_1,27_ = 6.08, p<0.03; Fig. 3c). In BAT, only exercise had a significant effect on CS activity (F_1,27_ = 142.68, p<0.0001), and thermal acclimation did not (F_1,27_ = 0.92, p = 0.35; Fig. 3d).

We determined lactate dehydrogenase (LDH) activity to assess whether there is a change in glycolytic, anaerobic ATP production. LDH activity was significantly higher in muscle of exercised rats compared to sedentary animals (F1_,35_ = 53.54, p<0.0001) but cold exposure had no effect (F_1,35_ = 0.061, p = 0.81; Fig. 3e). In BAT, neither exercise (F_1,35_ = 2.66, p = 0.11) nor thermal acclimation (F_1,35_ = 1.67, p = 0.21) affected LDH activity (Fig. 3f).

### Whole animal oxygen consumption

**Figure 4:**
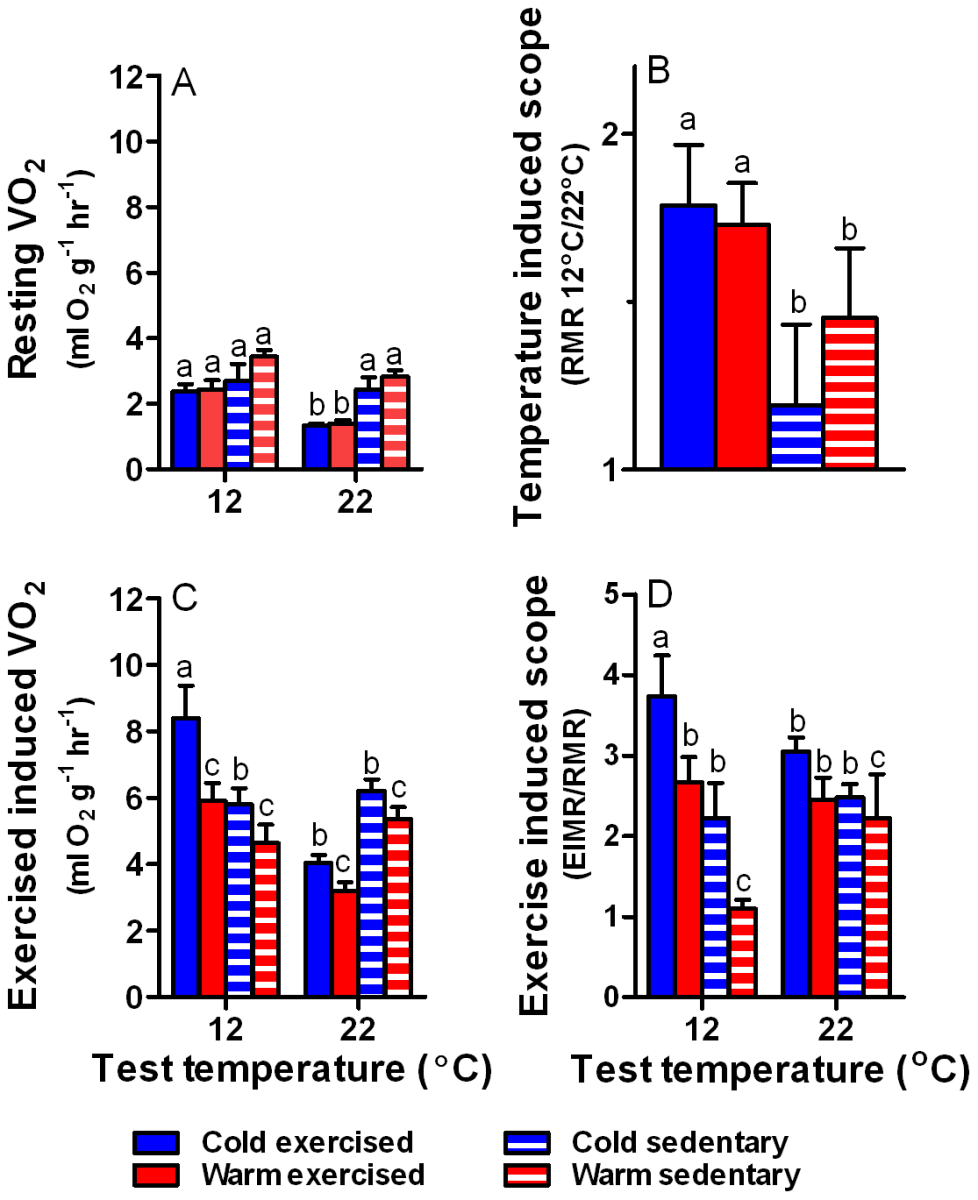
Oxygen consumption and metabolic scopes of cold and warm acclimated sedentary and exercised rats. Resting (RMR; A) and exercise induced (EIMR; C) metabolic rates (ml O_2_.g^−1^.hr^−1^) in cold exercised (blue bars), warm exercised (red bars), cold sedentary (blue hatched bars) and warm sedentary (red hatched bars) rats (mean ± s.e.m) measured at different ambient test temperatures (12°C and 22°C). Resting metabolic rates (A) were lowest in exercised animals at 22°C, and exercised induced metabolic rates (C) were highest in cold acclimated exercised rats. Temperature induced metabolic scope (RMR at 12°C/RMR at 22°C; B) was greatest in exercised rats, and exercise induced metabolic scope (EIMR/RMR; D) was greatest in cold acclimated exercised rats at 12°C. Letters above columns indicate significant differences; please note however that there were also significant interactions which are difficult to represent graphically.

Resting metabolic rate (RMR) increased with decreasing test temperature in exercised rats, and it was lower in exercised rats compared to sedentary rats at the higher ambient temperature of 22°C (exercise x test temperature interaction: F_1,22_ = 9,19, p<0.01; Fig. 4a). Temperature induced metabolic scope (RMR 12°C/22°C) was significantly greater in exercised rats compared to sedentary rats (F_1,25_ = 9.88, p<0.01) regardless of thermal acclimation (F_1,25_ = 0.001, p = 0.98; Fig. 4b). Exercise induced metabolic rate (EIMR) was significantly higher in cold acclimated rats compared to warm acclimated rats (F_1,30_ = 9.75, p<0.005). Importantly, exercise induced metabolic rate increased significantly in exercised rats at 12°C but not in sedentary rats (test temperature x exercise treatment interaction: F_1,30_ = 36.82, p<0.0001; Fig. 4c). Exercise induced metabolic scope (EIMR/RMR) was significantly greater in cold acclimated (F_1,21_ = 9.99, p<0.005) and exercised (F_1,21_ = 15.42, p<0.001) rats. Interestingly, at low temperature scope increased in cold acclimated and exercised rats but not in animals from other treatments (test temperature x exercise interaction: F_1,21_ = 4.99, p<0.05; Fig. 4d).

### Critical sustained running performance

**Figure 5:**
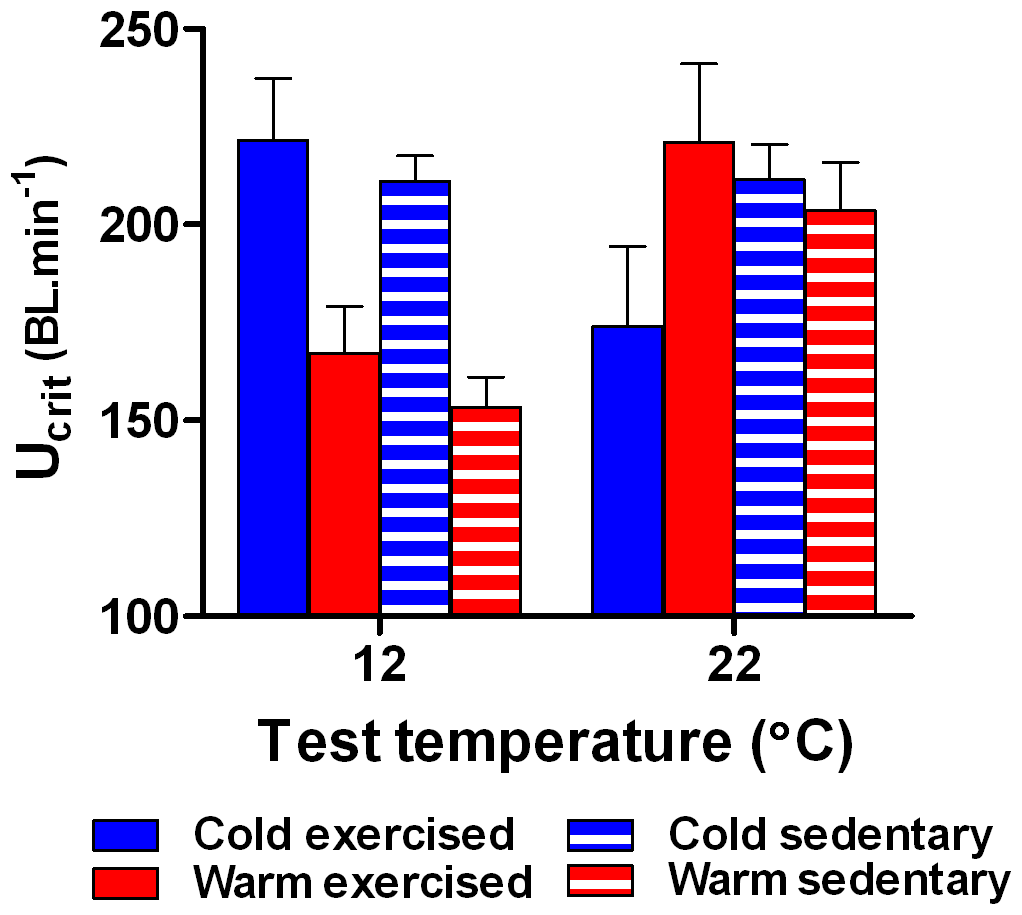
Critical sustained running speed of cold and warm acclimated sedentary and exercised rats. Critical sustained running speed (U_crit_) of cold exercised (blue bars), warm exercised (red bars), cold sedentary (blue hatched bars) and warm sedentary (red hatched bars) rats at 12°C and 22°C (mean ± s.e.m). There was a three-way interaction between exercise, thermal acclimation, and test temperature. Cold acclimated animals performed best, and warm acclimated animal performed worst at 12°C. Cold acclimated and exercised rats performed worst at 22°C.

Critical sustained running performance (U_crit_) varied with exercise, thermal acclimation and test temperature (three way interaction: F_1,31_ = 7.98, p<0.01), but none of the main effects was significant (all F_1,31_<2.3, p>0.14; Fig. 5). U_crit_ was lower in warm acclimated rats at 12°C regardless of exercise training, and cold acclimated, exercise trained rats performed worst at 22°C. There is no indication that our exercise regime had a training effect that improved running performance (i.e. no main effect of exercise F_1,31_ = 0.007, p = 0.936).

## Discussion

Physical activity at temperatures below the thermal neutral zone is the typical condition for non-dormant (torpid or asleep) animals. Here we show that physical activity and cold exposure interact in their effects on metabolism. Cold alone has no effect on transcriptional regulators and causes only a limited increase in mitochondrial enzyme activities. Coordination and quantitative adjustment of metabolic pathways depends on induction or repression of transcription of a small number of transcription factors and their coactivators [17], of which PGC-1α plays an overarching regulatory role [19]. Modulation of PGC-1α leads to a large number of downstream effects that include cardiovascular function and angiogenesis [28], [29], substrate utilisation and switching, including insulin sensitivity, glucose transport, and gluconeogenesis [30], [31], and muscle fibre differentiation [32]. Hence, our finding that routine physical activity is a necessary prerequisite for increases in PGC-1α expression in response to a second stimulus – cold in our case – has implications that extend beyond adjustments of metabolic rate. This role of exercise is emphasised by the fact that two principal targets of PGC-1α, NRF-1 and PPARδ, show similar expression patterns as PGC-1α in response to physical activity and cold, and that there is a significant correlation between PGC-1α relative expression and that of the other transcriptional regulators as well as UCP1. PGC-1α facilitates the interaction between NRF-1 and the mitochondrial transcription factor Tfam, which regulates mitochondrial DNA replication and transcription [17]. PPARδ plays a critical role in the transcriptional regulation of muscle metabolism and mitochondrial fatty acid catabolism [13], [23], [33]. Hence, at least in *R. fuscipes*, it appears that physical activity is a necessary prerequisite for increases in mitochondrial abundance and oxidative metabolism. Changes in the expression of transcriptional regulators are reflected in the increased activity of rate limiting enzymes of the tricarboxylic acid cycle and electron transport chain, as well as of glycolysis.

Cold can stimulate mitochondrial metabolism and abundance via the sympathetic nervous system, and adrenergic receptors on the cell surface can stimulate PGC-1α transcription via cAMP and CREB signalling [19]. Similalry, excess caloric food intake is sensed by the brain, which will mediate an increase in energy expenditure via adrenergic receptors [34]. The similar sympathetic pathways that induce energy expenditure in response to cold and caloric intake suggest that the latter response may also depend on a minimum of physical activity, although this suggestion must be verified experimentally. Exposure to temperatures below the thermal neutral zone effectively increases energy expenditure [16], [35] that could counteract obesity. However, we show that such a response will be ineffective in the absence of regular physical activity, and that the temperature induced metabolic scope is significantly reduced in sedentary animals.

PGC-1α also regulates non-shivering thermogenesis in brown adipose tissue [20], [27] by inducing the expression of uncoupling protein 1 (UCP1). UCP-1 facilitates non-shivering thermogenesis in BAT by uncoupling electron transport in the inner mitochondrial membrane from oxidative phosphorylation [2], [27]. Interestingly, UCP-1 relative gene expression increased only in animals that exercised regularly. Hence, any BAT mediated increase in energy expenditure also relies on regular physical activity. As a corollary, adaptive thermogenesis in sedentary individuals living in more extreme environments may also be limited because of reduced UCP-1 expression, and because of the decreased mitochondrial capacity and abundance. The differences between treatment groups were pronounced even in our experimental environments that were relatively benign, and which did not compromise thermoregulation [36]. We did not aim to push animals to their physiological limits as is often done when testing thermogenic capacity of mammals; rather, our data have important implication for day-to-day (routine) energy expenditure. Animals including humans live mostly below their thermal neutral zone, so that ambient temperature will act as a stimulator for metabolic activity and energy expenditure most of the time. For example, the thermal neutral zone of *R. fuscipes* in 29–31°C (Glanville, unpublished data), which the animals almost never experience in the wild [37]. Similarly, the thermal neutral zone of humans is 33–35°C [38], which is rarely experienced except maybe at low latitudes. We show that although decreasing ambient temperature can potentially trigger energy expenditure, the responsiveness to ambient temperature changes is blunted in the absence of regular exercise. Hence, exercise is beneficial by directly causing expenditure of energy, and by enabling ambient temperature induced energy expenditure. One caveat to this conclusion is that sedentary animals had a higher metabolic rate at rest and at warm ambient temperatures (22°C) than exercised rats. However, with physical activity and a decrease in ambient temperatures metabolic rates increased above all other conditions, which confirms our hypothesis that physical activity and cold have an interactive effect on metabolic rate and energy expenditure. This conclusion is further confirmed by the fact that rats from the cold and exercise treatment had significantly greater food intake compared to all other treatments without, however, changing their body mass (36).

Both of our acclimation treatments were below the thermal neutral zone of the species, which mean that rats even in our warm (22°C) treatment may have had increased metabolic capacities compared to minimum levels at temperatures within the thermal neutral zone. Nonetheless, the muted response of sedentary rats to a further decrease in acclimation temperatures from 22°C to 12°C is extraordinary, both with respect to the implications discussed above and with respect to the consensus in the literature that cold stimulates metabolic responses, particularly in BAT (19, 27). Our acclimation and test temperatures were purposely moderate, and cold-induced metabolic responses in the laboratory are commonly observed at much lower ambient temperatures (<5°C; e.g. 19). More extreme cold exposure will stimulate uncoupling proteins and BAT oxidative capacity in the absence of physical activity (26, 27). Responses of BAT to very low temperatures alone are adaptive for animals that have to cope with extreme cold, and it suggests that there may be a threshold of activation below which exercise becomes relatively unimportant for BAT activation. This latter suggestion is speculative, but provides the basis for an interesting and feasible hypothesis to test.

There was no training effect on locomotor performance as a result of the exercise regime we imposed on the animals. Hence, metabolic responses to exercise do not require intensive exercise or training regimes that lead to increased locomotor performance. Thermal acclimation had a greater effect on locomotor performance than exercise, which is in contrast to the metabolic responses we report and indicates that running at this level is not constrained by metabolic capacity.

### Conclusions

Regular physical activity not only increases energy expenditure directly, but also modulates gene expression patterns of metabolic regulators. Gene expression patterns induced by a sedentary lifestyle result in metabolic patterns that are the precursors of metabolic diseases prevalent among humans [6], [8], [39]–[40]. For example, decreased PGC1α expression results in increased insulin resistance, predisposing an animal to obesity and type 2 diabetes [15]. Impaired glucose and fatty acid metabolism due to decreased PPARδ expression also increases the risk of obesity and type 2 diabetes [24], [39]–[40]. The deletion of PPARδ results in increased fat accumulation in the heart and decreased survival in mouse models [41]. Additionally, decreased concentrations of glucose transporter 4 (GLUT4) as a result of reduced NRF-1 expression in sedentary animals leads to a fall in glucose transport which predisposes individuals to type 2 diabetes [21], [42].

When combined with cold exposure, exercise promotes uncoupling of oxidative phosphorylation by increasing the expression of UCP1 in BAT. Increased expression of UCP1 increases the proportion of energy derived from fatty acid oxidation that is released as heat, rather than being deposited as white adipose tissue; this provides an important avenue by which exercise facilitates increased energy expenditure and counteracts obesity [43]. Obesity, although complicated by social and cultural influences, is due to an excess of energy intake over energy expenditure [4]. In mouse models, over-expression of UCP1 results in obesity resistance [44]–[45] while ablation of UCP1 induces obesity [46]. Brown adipose tissue therefore plays an important role in the energy balance of the whole organism including adult humans [47], [48], but we show that exercise is a necessary condition for this role to be realised. A practical implication of our results is that research on metabolism using sedentary animals is likely to produce results that are limited in their generality, and which cannot be transferred to more ecologically relevant contexts.

## Materials and Methods

### Ethics Statement

All experimental procedures were approved by the University of Sydney Animal Ethics Committee (permit L04/12-2006/3/4512) and the NSW National Parks and Wildlife Services (Scientific License S12186).

### Experimental animals and acclimation treatments

We used a wild rodent, the bush rat *Rattus fuscipes*, as a model to avoid potential problems from inbreeding and thereby altered exercise responses in laboratory strains. Bush rats were trapped near Sydney, Australia (33°42′41″S, 151°13′58″E) and kept individually in plastic cages (350×220×220 mm). We used only adult animals, and excluded any pregnant females; all experimental groups had a even sex ratio. Rats were fed ad libitum on a mixture of commercial rodent mix and diced apples. Median body mass of experimental animals was 115.0 g and body mass did not change significantly during the acclimation treatments in any experimental group. For more information on food intake and body mass of the animals please see [36].

After a 14 day habituation period, rats were randomly allocated to either a cold (12°C) or a warm (22°C) acclimation treatment with a constant photoperiod of 12L:12D in all treatments; acclimation treatments lasted for 30 days. The rationale for our treatments was to expose animals to conditions naturally and routinely experienced rather than testing physiological limits under extreme conditions. Hence, acclimation temperatures were based on mean winter and summer temperatures naturally experienced by the population from which the rats were sampled [37]. Please note, however, that even the “warm” experimental conditions are below the thermal neutral zone of the animals. Within each thermal acclimation group, rats were allocated to either a sedentary (n = 10 cold and n = 10 warm) or an exercise (n = 8 cold and n = 8 warm) treatment. We took especial care to minimise handling of the animals, and to treat all experimental animals the same during the acclimation period to minimise any potential confounding effects of handling.

Rats in the exercise treatments were run in a wheel on five days per week throughout the acclimation period. In each exercise session, bush rats were placed individually into an enclosed, motorised running wheel for 30 minutes at 60% of the running speed that resulted in exercise induced metabolic rate as determined in preliminary studies (according to methods described in Locomotor performance below). Bush rats were euthanased by an overdose of sodium pentabarbitone (120 mg kg^−1^ i. p.) on a day following an exercise day, but the animals were not exercised on the day of euthanasia.

### Gene expression

Skeletal muscle and BAT samples were collected at the time of euthanasia and immediately stored in RNAlater (Ambion, USA) at −20°C. RNA extraction and reverse transcription were conducted according to published protocols [49]. Quantitative RT-PCR was performed on an Applied Biosystems 7500 qRT-PCR machine (Applied Biosystems, Foster City, USA). Commercial gene expression assays for *Rattus norvegicus* (TaqMan, Applied Biosystems) were used for analyses of PPARδ, PGC-1α and NRF-1 mRNA concentrations according to the manufacturer's instructions.

Primers were designed from sequences obtained from GenBank for *Rattus norvegicus* uncoupling-protein 1 [GenBank: NM012682; forward: gactcggatcctggaacgtc; reverse: gcataggagcccagcatagg] and nuclear 28S RNA [GenBank: V01270; forward: gcctcacgatccttctgacc; reverse: aacccagctcacgttcccta]. For these genes we used real-time PCR reactions with SYBR green that contained 1× SensiMixPlus SYBR (Quantace, United Kingdom), 4.5 mM MgCL_2_, 600 nM primer and 50 ng cDNA. The cycle consisted of 95°C for 7 min, 40 cycles of 95°C for 15 s and 66°C for 1 min; 95°C for 15 s; 60°C for 1 min, and 95°C for 15 s. We performed melt curve analysis to ensure that only a single PCR product was amplified, and all assays were run in duplicate. Relative gene expression of the target gene was calculated according to [50] with the nuclear ribosomal 28S RNA gene as reference gene and the grand mean of all treatments as control; in the text we refer to ‘relative expression’ in the sense of [50].

### Enzyme activities

Skeletal muscle (vastis lateralis) and BAT were collected at the time of euthanasia and were transferred into liquid nitrogen immediately after collection and stored at −80°C for later analysis of lactate dehydrogenase (LDH), cytochrome c oxidase (COX) and citrate synthase (CS) activities. Enzyme activities were determined according to published protocols [51] at 37°C [36].

### Whole animal oxygen consumption

Standard flow through respirometry was used to measure whole animal rates of oxygen consumption at 12°C and 22°C [52]. Rats rested at the test temperature in the metabolic chambers (100×80×120 mm) for 2 h before measurements of resting oxygen consumption; animals were observed with a remote camera during measurements. Oxygen consumption was measured with a gas analyzer (ML206, ADInstruments) connected to a computerised recording system (PowerLab; ADInstruments). Resting metabolic rate of all rats was measured at each temperature in a random order. Design, calibration and use of the system followed published protocol with room air (20.95% O_2_) and pure N_2_ (0% O_2_) used for calibration of the gas analyser [52]. Carbon dioxide was scrubbed from the air using granules of Baralyme before entering the chamber and from subsampled gas prior to analysis. Water was absorbed from the air before entering the chamber with Drierite and after the chamber using a drying tube (MLA0343, ADInstruments). Air was pumped through the chamber at a rate of 600–700 mL.min^−1^. Gas sampling rates were set at 100 mL.min^−1^, and flow rates were measured with a flowhead (MLT10L, ADInstruments) connected to a spirometer (ML141, ADInstruments) and the PowerLab system (ADInstruments). When oxygen consumption had plateaued, generally after 2 hours, resting metabolic rate was determined as the mean of the five lowest consecutive oxygen consumption measurements. Mass specific oxygen consumption (mL. O_2_. hr^−1^. g^−1^) was calculated using the standard equation V_O2_ = V_1_ (F_1_O_2_-F_e_O_2_)/(1-F_e_O_2_) where V_1_ is the inlet air flow rate (ml.h^−1^), F_1_O_2_ is the inlet fractional O_2_ content (0.2095) and F_e_O_2_ is the excurrent fractional O_2_ content [52]. Measurements were made in a temperature controlled room (±0.5°C), and the temperature within the chamber was measured with a calibrated thermocouple connected to a PowerLab (ADInstruments). The animals were post-absorptive and in their resting phase during the measurements. For all oxygen consumption measurements, reference samples were taken before and following the trials. Exercise induced metabolic scope was calculated by dividing exercise induced metabolic rate (see Locomotor performance below) by resting metabolic rate. Temperature induced metabolic scope was calculated by dividing resting metabolic rate at 12°C by resting metabolic rate at 22°C.

### Locomotor performance

Critical sustained running speed (*U*_crit_) was measured at 12°C and 22°C in ramped speed trials in a motorised running wheel fixed within a Perspex metabolic chamber (220×120×240 mm), which also allowed measurements of oxygen consumption (see oxygen consumption above). Wheel speed was controlled by a motor regulated with a DC power supply (MP3090; Powertech, Osborne Park, WA, Australia) and rotations were counted with a cyclometer calibrated to the circumference of the running wheel. The settings on the power supply were calibrated for wheel rotations (km.h^−1^) so that running speed could be controlled by adjusting the DC power input. The running wheel was placed in a temperature-controlled room and the temperature within the wheel was monitored with a calibrated thermocouple connected to a computerised recording system (Powerlab, AD Instruments, Sydney, Australia).

Critical sustained running speed was determined as *U*_crit_ = *U*_f_+[(*t*_f_/*t*_i_)*U*_i_], where *U*_f_ is the greatest running speed maintained for a whole time interval, *t*_f_ is time spent at the final speed, *t*_i_ is the time interval between speed increments and *U*_i_ is the speed increment [53]. Pilot studies were performed to determine *t*_i_ (150 s), *U*_i_ (0.25 km.h^−1^) and initial running speed (0.75 km.h^−1^). Animals were placed individually in the wheel and allowed to habituate at the test temperature for 2 h. All but one rat in the warm-exercised treatment ran in the wheel. Animals were run until fatigued, which was defined as the time when animals could no longer maintain position in the wheel. Each rat was run at 12°C and at 22°C in random order, with a minimum of 24 h between runs.

Oxygen consumption was measured during the running trials to give exercise induced metabolic rate at 12°C and 22°C. Oxygen consumption was measured using the same set-up as described above for resting metabolic rate. However, for measurements of exercise induced metabolic rate the flow rate was increased to 1400 mL.min^−1^. Exercise induced metabolic rate was calculated as the highest instantaneous VO_2_ averaged over 1 min intervals [54].

### Statistical analysis

We analysed relative expression of target genes [50], enzyme activities, and temperature induced metabolic scope by 2-way analyses of variance (ANOVA), with thermal acclimation and exercise as independent, fixed factors. Resting and exercise induced metabolic rate and scope, and sustained running speed were analysed by 3-way ANOVA with thermal acclimation and exercise as independent, fixed factors, and ambient test temperature at which trials were performed as repeated measure. We used Levene's test to test for homogeneity of variances, and data were log transformed in case of heteroscedacity.

To explore relationships between PGC-1α and its target genes, we performed regression analyses between PGC-1α relative expression and that of PPARδ, NRF1, and UCP1 across all treatments. All data are expressed as means + s.e.m. and the level of significance was α = 0.05.

We used the truncated-product method [55] to assess the effect of multiple hypotheses testing on the validity of p values. Briefly, the truncated-product method considers the distribution of p values from multiple hypothesis tests to provide a table-wide p value for the overall hypothesis that significant results in the set were truly significant rather than due to chance. Multiple comparisons did not bias the statistical results presented here (p<0.001).
